# Influence of a major pre‐clinical programme restructure on undergraduate student fixed prosthodontics clinical unit completions

**DOI:** 10.1111/eje.12791

**Published:** 2022-02-24

**Authors:** James Dudley

**Affiliations:** ^1^ Adelaide Dental School The University of Adelaide Adelaide South Australia Australia

**Keywords:** clinical unit completion, fixed prosthodontics, pre‐clinical simulation teaching, undergraduate curriculum

## Abstract

**Introduction:**

Research is limited in measuring the effectiveness of pre‐clinical programmes in preparing students for fixed prosthodontics clinical practice. The aim of this retrospectively study was to assess the influence of a major pre‐clinical programme restructure on undergraduate student fixed prosthodontics clinical unit completions.

**Materials and Methods:**

The fixed prosthodontics treatment registers from 2011 to 2020 were reviewed, and units completed per student (UCS) and units completed per student per session (UCSS) were calculated in the years before (2011–2013) and after (2014–2020) a major pre‐clinical programme restructure (PR). Data were summarised in Microsoft Excel software (version 2016), and Student's *t*‐test and paired *t*‐tests were performed to determine the significance of difference in UCS and UCSS in the years before and after the PR.

**Results:**

There was a significant difference in the UCS (*p* < .05) and UCSS (*p* < .01) in the years before and after the PR. The average UCS in the years before the PR was 2.20 units compared with 3.86 units after the PR, an increase of 75% per student. The average UCSS in the years before the PR was 0.15 units compared with 0.28 units after the PR, an increase of 87% per session.

**Conclusion:**

The fixed prosthodontics pre‐clinical programme restructure resulted in statistically significantly increased student clinical unit completions.

## 1 INTRODUCTION

The teaching of fixed prosthodontics to undergraduate dental students has traditionally commenced with pre‐clinical practical components conducted in a variety of settings ranging from benchtops to dental manikin simulations to virtual simulations. Typically, students have opportunities to develop practical skills in a simulation environment by completing formative exercises leading to a summative practical assessment.^1^, ^2^ The subsequent transition to clinical practice remains a challenge^3^; however, the importance of developing practical skills has been reinforced by the positive correlation of fixed prosthodontics pre‐clinical grades with clinical grades in operative dentistry and fixed prosthodontics.^4^

Fixed prosthodontics techniques are generally considered challenging and require understanding of core concepts and demonstration of a high degree of manual dexterity. Therefore, it is important to graduating dentists who have sound skills and experience in complex restorative dentistry and prosthodontics techniques in preparation for clinical practice.

The use of dental manikin simulation clinic teaching has replaced significant components of clinical practice in dental curricula around the world^1^ and provides teaching efficiency and effectiveness gains and improved convenience for teachers and students.^5^ As techniques have evolved, the benefits of teaching alternative and modernised fixed prosthodontics techniques have been established in producing closer to ideal crown preparations.^6^, ^7^ Furthermore, dental simulation teaching has expanded beyond the manikin model to encompass virtual reality technologies.^1^, ^8^, ^9^

Artificial teeth have been used in fixed prosthodontics pre‐clinical simulation teaching for many years. The use of artificial teeth provides benefits including standardisation of teeth, standardisation of teaching techniques, facilitation of repeatable practical demonstrations, life‐like appearance and being readily available.^8^ The limitations include variations in the accuracy of anatomically simulating natural teeth, different surface textures and the cost involved in obtaining teeth, models and manikins.^8^ Nevertheless, artificial teeth have become a mainstay in fixed prosthodontics pre‐clinical teaching.

At this university, fourth‐year undergraduate dental students undertake a six‐week intensive fixed prosthodontics pre‐clinical programme that provides a comprehensive and intensive simulation clinic experience in crown preparations, temporary crowns and post‐core techniques involving structured and sequenced theoretical and practical elements. The programme commences with simple fixed prosthodontics procedures and progresses to more complex techniques and culminates in an end‐of‐programme assessment.

At the end of 2013, the fixed prosthodontics pre‐clinical programme was completely rewritten, renewed, reconfigured, expanded and resequenced in accordance with a peer review of teaching and an evidence‐based approach, and relocated from a “bench‐top” setting to a purpose‐built simulation clinic where all exercises were conducted in dental manikins, hereafter termed the programme restructure (PR). The refreshed and modernised programme provides a high‐fidelity experience in fixed prosthodontics. The teaching is structured and sequenced, and each topic involves a flipped classroom pedagogical approach reinforced by relevant journal article readings, demonstration videos, small group supervised practical sessions guided by specific and detailed procedural instructions, and skill building throughout each technique exercise culminating in formative assessment of each exercise. The range of techniques include porcelain‐bonded to metal crown preparations, porcelain‐bonded to zirconia crown preparations, full gold crown preparations, ceramic veneer preparations, gold and ceramic on lay preparations, porcelain‐bonded to metal bridges, metal alloy bridges, direct and indirect post‐cores, and temporary restorations for all the listed procedures. After the PR, the pre‐clinical programme was maintained in its restructured format.

Satisfactory completion of the programme exercises is required to qualify for the end‐of‐programme assessment that assesses pre‐clinical competency. The assessment involves completion of crown preparations and temporary crowns within a time limit, student self‐assessment of the completed work and blinded marking against marking rubrics conducted by experienced teaching staff. One redemption opportunity is provided. Remediation is provided for students who do not attain a satisfactory standard or who need additional practice. On achieving a satisfactory pre‐clinical standard, students commence clinical treatment of patients in supervised sessions throughout an academic year.

One measure of student performance throughout each clinical year is unit completions, where a unit is defined as any fixed prosthodontic procedure requiring laboratory fabrication. Throughout the academic year, fourth‐year dental students record their completed units in fixed prosthodontics treatment registers, which are continually updated and reviewed and co‐signed by the supervising clinical tutor. The registers provide an accurate representation of clinical unit completions, and at the end of the academic year, the unit totals are summarised and reviewed by the discipline coordinator. Unit completions receive formative assessment feedback recorded on the treatment registers. At the end of each semester, clinic summative assessments are conducted against detailed set assessment criteria in accordance with contemporary dental education philosophy; however, this aspect of student performance did not form part of the current study.

The pre‐PR pre‐clinical programme had a restricted scope of experience, used dated teaching methods and had limitations in preparing students for clinical practice. It was observed that significant clinical time was used to educate essential procedures, which restricted unit completions. The aim of this retrospectively study was to assess the influence of a major pre‐clinical programme restructure on undergraduate student fixed prosthodontics clinical unit completions.

## 2 MATERIALS AND METHODS

### 2.1 Data collection and analysis

The fixed prosthodontics treatment registers from 2011 to 2020 were reviewed, and total number of students per year, student gender, total units per year and total number of clinic sessions per year were recorded. The average units completed per student (UCS) and average units completed per student per session (UCSS) were calculated for each year in Microsoft Excel software (version 2016) and presented in tabulated and graph form. The pre‐PR years were 2011–2013, and the post‐PR years were 2014–2020.

Student's *t*‐test was performed to determine whether there was a significant difference in UCS and the UCSS in the years before and after the PR. Paired *t*‐tests were performed to determine whether there was a significant difference in the UCS and UCSS in each year before and after the PR.

Microsoft Excel software (version 2016) was used, and the level of significance was set at *p* = .05.

## 3 RESULTS

**FIGURE 1:**
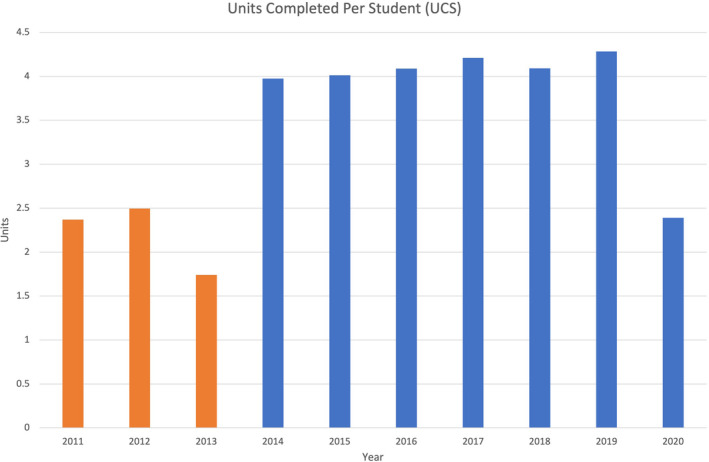
Units completed per student (UCS)—orange bars before PR; blue bars after PR

**FIGURE 2:**
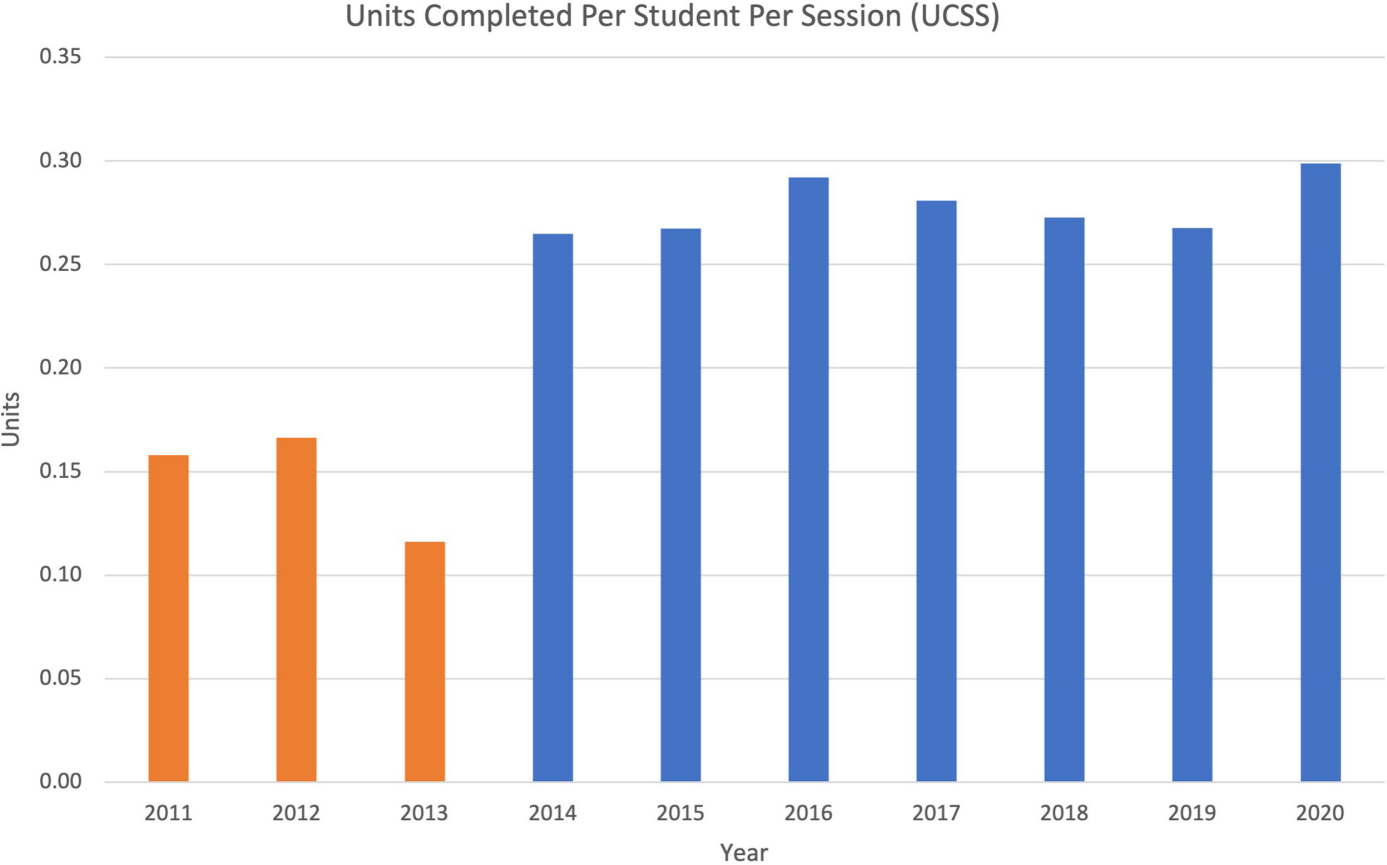
Units completed per student per session (UCSS)—orange bars before PR; blue bars after PR

The total number of students per year and gender distribution is provided in Table 1. The UCS and UCSS data are presented in Figures 1 and 2. There was a significant difference in the UCS and UCSS in the pre‐PR years and post‐PR years (UCS: *t* = 3.99, *p* < .05; UCSS: *t* = 10.33, *p* < .01).

**TABLE 1:** Students per year and gender distribution

Year	Male students	Female students	Total students
2011	33	48	81
2012	37	50	87
2013	30	51	81
2014	33	43	76
2015	40	47	87
2016	33	47	80
2017	26	45	71
2018	29	48	77
2019	28	43	71
2020	29	41	70

The average UCS in the pre‐PR years was 2.20 units compared with 3.86 units in the post‐PR years, an increase of 75% per student. Excluding the COVID‐affected 2020 year, the average UCS post‐PR was 4.11 units. The average UCSS in the pre‐PR years was 0.15 units compared with 0.28 units in the post‐PR years, an increase of 87% per session. There was a significant difference in the UCS and UCSS in each post‐PR year compared with each pre‐PR year (UCS: *t* = 12.34, *p* < .01; UCSS: *t* = 22.23, *p* < .01).

The 2020 academic year was affected by COVID‐related clinic closure which resulted in half the normal number of clinic sessions per year. The variations in UCS (Figure 1) within the pre‐PR years and also within the post‐PR years were not significantly different to one another (pre‐PR: *t* = 0, *p* = 1; post‐PR: *t* = .10, *p* = .92) with the exception of the UCS in 2020 (when compared to the other post‐PR years), which was significantly different (*t* = 5.73, *p* < .01) due to the reduced number of clinic sessions in the year.

## 4 DISCUSSION

There was an average UCSS increase of 87% observed in the years following the PR. The average UCS increase of 75% was different to the average UCSS increase and can be reasoned by the UCS analysis not allowing for the COVID‐related reduction in the number of clinical sessions in the 2020 clinical year. The significant improvement in productivity, efficiency and greater clinical experience after the PR was maintained consistently across the post‐PR years. The 10 year study period, during which the post‐PR was maintained, permitted reliable and definitive conclusions to be made.

Fixed prosthodontics pre‐clinical teaching for students is provided to deliver educational information, develop procedural understanding and facilitate skill development. The programme is designed to bridge the gap between the end of the third year (when there is minimal fixed prosthodontics practical experience) and the commencement of clinical practice on selected patients. The pre‐PR programme involved a two‐week overview conducted on “benchtops” involving “demonstration then do” for simple techniques. The programme was largely unstructured with completion of the exercises the only requirement for commencing clinical practice.

The post‐PR renewed programme was comprehensive and involved intensive tuition at a 1:10 tutor‐to‐student ratio, continual formative assessment, individual review meetings, an end‐of‐programme summative assessment and an associated remedial programme. All practical exercises were conducted in a purpose‐built simulation clinic in dental manikins. The teaching content was modernised and aligned with modern fixed prosthodontics philosophies. Each practical session was structured with an interactive, blended learning, flipped classroom approach with sequenced didactic teaching, journal article reading, step‐by‐step procedural guides, demonstration videos and culminating with the practical exercises. The practical exercises commenced at a basic standard on more easily accessed and prepared teeth and provided multiple opportunities for students to develop their skills in the same technique before proceeding to the next exercise. The formative assessment involved student self‐evaluation of work, supervising tutors providing verbal and written feedback and review meetings with the course coordinator to evaluate progress where successes were recognised and areas for improvement identified. The end of course summative assessment involved completion of crown preparations and temporary crowns within a time limit, student self‐assessment of the completed work and blinded marking against marking rubrics conducted by experienced teaching staff, with one redemption opportunity offered. The use of a defined end‐point practical assessment was a key component in establishing a minimum level of student competency prior to the commencement of safe clinical treatment of patients.

The positive correlation of fixed prosthodontics pre‐clinical grades with clinical grades has been reported but reasons for the observations have not been explored.^4^ The link between skill building in pre‐clinical programmes and clinical performance in fixed prosthodontics is critical not only in the student educational experience but also in maintaining standards and ensuring patient safety. It is important to note that final clinical assessments do not solely rely on unit completions, but also include performance measured against set clinical assessment criteria nested within formative and summative assessment processes. In an environment of limited clinical experience where in the post‐PR years, an average of 3.86 units (or 4.11 units excluding the COVID‐affected 2020 year) were completed per student in the clinical year, greater clinical experience through managing a larger number of units can only be beneficial for student experience.

There are limited direct comparative studies as this is the first known research of its kind in this specific field of investigating the influence of a comprehensive pre‐clinical programme restructure on undergraduate student fixed prosthodontics clinical unit completions. A related study investigated the effectiveness of a fixed prosthodontics course in preparing students to treat patients in the clinic, but this study relied on the student report of experience which brings into consideration many potential confounding student factors.^10^ Student experience of learning and teaching is an important measure of programme evaluation^11^, ^12^; however, the student view may not necessarily reflect the educator's view.^13^ Students may view more difficult (but required and beneficial) exercises as less enjoyable, they may be influenced by their educators in their satisfaction of the course, and students have entry‐level experience and are limited to the student side of the educational process. The present study does not report on student experience of learning and teaching, rather it uses clinical unit completion data, which is a separate measure and is limited to assessing productivity.

The pre‐clinical programme was a precursor to the clinical programme; therefore, the observed results in the present study were associative. However, the clinical environment, group sizes, clinic session duration, clinical assessment philosophies, patient supply, method of measuring units and laboratory support remained the same over the study duration; there were no clinical factors that changed.

The current study was conducted at a single dental school, and the results are representative of the student cohorts at this teaching institution and may not be generalisable to students at other universities. All data were retrospective and were analysed at a single point in time after the completion of each of the included academic years.

A range of factors could have contributed to the observed results, and in variable proportions. There may have been more dominant factors that are not possible to individualise and measure as this was a multi‐faceted PR. For example, the move to a brand new purpose‐built simulation clinic could have had a significant positively motivational effect on the students. Completing the pre‐clinical exercises in the simulation clinic manikin instead of on the benchtop may have improved student technical skills as has been proposed but not proven in a previous study.^14^

Despite these considerations and in the context of limited previous research in this area, this study's findings provided an encouraging account of the effectiveness of the PR and serve to nurture further research in this area. It was the author's observation that the PR better prepared the students for clinical practice through more rapid upskilling and improved efficiencies of practice allowing more fixed prosthodontics procedures to be completed more often in a single session resulting in more fixed prosthodontics clinical units.

## 5 CONCLUSION

The fixed prosthodontics pre‐clinical programme restructure resulted in statistically significantly increased student clinical unit completions.

## ETHICS APPROVAL AND CONSENT TO PARTICIPATE

Ethics approval was obtained from the Human Research Ethics Committee at The University of Adelaide.

## CONFLICT OF INTEREST

The author declares there are no competing interests.

## AUTHOR CONTRIBUTIONS

There was a sole author who produced this work.

## Data Availability

Data are available from the author on request. The data are not publicly available due to privacy or ethical restrictions.
